# Tentative Characterization of Polyphenolic Compounds in the Male Flowers of *Phoenix dactylifera* by Liquid Chromatography Coupled with Mass Spectrometry and DFT

**DOI:** 10.3390/ijms18030512

**Published:** 2017-03-02

**Authors:** Ridha Ben Said, Hamed Arafa I., Mahalel Usam A., Al-Ayed Abdullah Sulaiman, Mariusz Kowalczyk, Jaroslaw Moldoch, Wieslaw Oleszek, Anna Stochmal

**Affiliations:** 1 Department of Chemistry, College of Science & Arts at Al-Rass, Qassim University, P.O. 53, 51921 Al-Rass, Saudi Arabia; asaaied@qu.edu.sa; 2 Physico-Chimie des Matériaux à l’état condensé, Department of Chemistry, Faculty of Sciences, University Tunis El Manar, 2092 Tunis, Tunisia; 3 Department of Biochemistry and Crop Quality, Institute of Soil Science and Plant Cultivation, State of Research Institute, ul. CzatRoryskich 8, 24-100 Pulawy, Poland; mkowalczyk@iung.pulawy.pl (M.K.); jmoldoch@iung.pulawy.pl (J.M.); wo@iung.pulawy.pl (W.O.); asf@iung.pulawy.pl (A.S.); 4 Phytochemistry Laboratory, Department of Botany, Faculty of Science, Aswan University, 81528 Aswan, Egypt; mahalel71@yahoo.com

**Keywords:** *Phoenix dactylifera*, electrospray ionization, mass spectrometry, *p*-coumaroylshikimic acids, caffeoylshikimic acids, Density Fonctional Theory (DFT)

## Abstract

*Phoenix dacylifera* is an ancient palm species rich in (poly)phenols. These phenolic compounds were tentatively identified by using liquid chromatography coupled with ion spray mass spectrometry in tandem mode (LC/MS/MS) with negative ion detection. Negative identification of the compounds was based on their retention times and mass spectra in full scan mode (MS), and in different MS/MS modes. For the first time, complete hypothesis, and routs for both *p*-coumaroylshikimic acids (CoSA) and caffeoylshikimic acids (CSA) were suggested and confirmed by Density Fonctional Theory (DFT) study. Notably, of the 53 compounds characterized, 19 hydroxycinnamates derivatives were tentativelycharacterized in male flowers of date palm and 15 of them were recorded for the first time. In addition, five organic acids, six B-type proanthocyanidins, two anthocyanidin and 21 flavonoid derivatives have been tentatively characterized. Identification of B-type proanthocyanidins were based on the diagnostic ions resulting from heterocyclic ring fission (HRF) and retro-Diels-Alder (RDA) reaction of flavan-3-ol provided information on the hydroxylation pattern and the type of inter-flavan bond proanthocyanidins. The sequence of proanthocyanidins was detected through ions extracted from quinone methide (QM) cleavage of the inter-flavan bond.

## 1. Introduction

*Phoenix dactylifera* (date palm) is a native to the Middle East region over centuries, cultivated for its edible sweet fruit. Date palm male flowers and seeds are widely used in the Arab region as a tonic drink as well as an anti-diabetic [1] and anti-oxidant [2,3,4]. *P. dactylifera* male flowers suspension is a herbal mixture that is widely used as a folk remedy for curing male fertility in traditional medicine [5,6]. In ancient Egypt, it was used to promote women’s fertility. The male flowers of the date palm are also eaten directly by people as a fresh vegetable, allegedly to enhance fertility. The previous study displayed that the extract of date palm pollen grains contains estrogenic compounds, estrone, as gonad-stimulating agents that improve male infertility and display gonadotropin activity in the rat [7]. The previous phytochemical studies on the Egyptian palm male flowers indicated the presence of cholesterol, diosgenin, estrone, estradiol, esteriol, β-amrin, β-sitosterol, and many flavonoids [8].

Hydroxycinnamates such as *p*-coumaric acid, caffeic acid, ferulic acid, syringic acid, sinapic acid, gallic acid and protocatechuic acid are widely spread throughout the plant families, which are combined with malic acid, anthranilic acid, shikimic acid, quinic acid and glycerol [9,10]. The isomers of caffeoylshikimic acids (3-, 4- and 5-CSA) were isolated from date of *P. dactylifera* as an enzymic browning substrate in dates [11]. 5-CSA was detected as the major hydroxycinnamoylshikimic acid derivative, and the 3- and 4-isomers were detected as minor components, no traces of *p*-coumaroylshikimic acids were detected [12].

Proanthocyanidins (PACs) are polymeric products of the flavonoid biosynthetic pathway with synonym name condensed tannins that represent one of ubiquitous groups of all plant phenols. The degree of polymerization can be used to describe the PAC molecules size [13]. PACs containing catechin, gallocatechin or afzelechin as subunits are named proanthocyanidin. PACs are found in many plant parts such as fruits, seeds, leaves and barks where they protect them against predation. Moreover, they give astringency to beverages and flavor such as fruit juices, wine and teas, and as having advantageous effects on human health.

Due to the difficulties of separation and structural complexity of hydroxycinnamates and proanthocyanidin derivatives, investigation on these compounds, in comparison with other polyphenols are limited [14,15]. Due to large number of phenolic groups and the resemblance in catechin structures, the HPLC peaks of catechin derivatives are usually unresolved [15,16]. RP-HPLC is the most communal method used in the analysis of these compounds [17].

To date, little phytochemical studies were reported for *P. dactylifera* male flowers [18]. The present work is focused on the analysis of the phenolic constituents of *P. dactylifera* male flowers as a novel rich source of phenolic acids, flavonoids and proanthocyanidins. To understand the variation of phenolic acid stereoisomers proportions, a theoretical calculation based on the Density Fonctional Theory (DFT) approach implemented in the GAUSSIAN09 series of programs were carried out.

## 2. Results and Discussion

**Figure 1:**
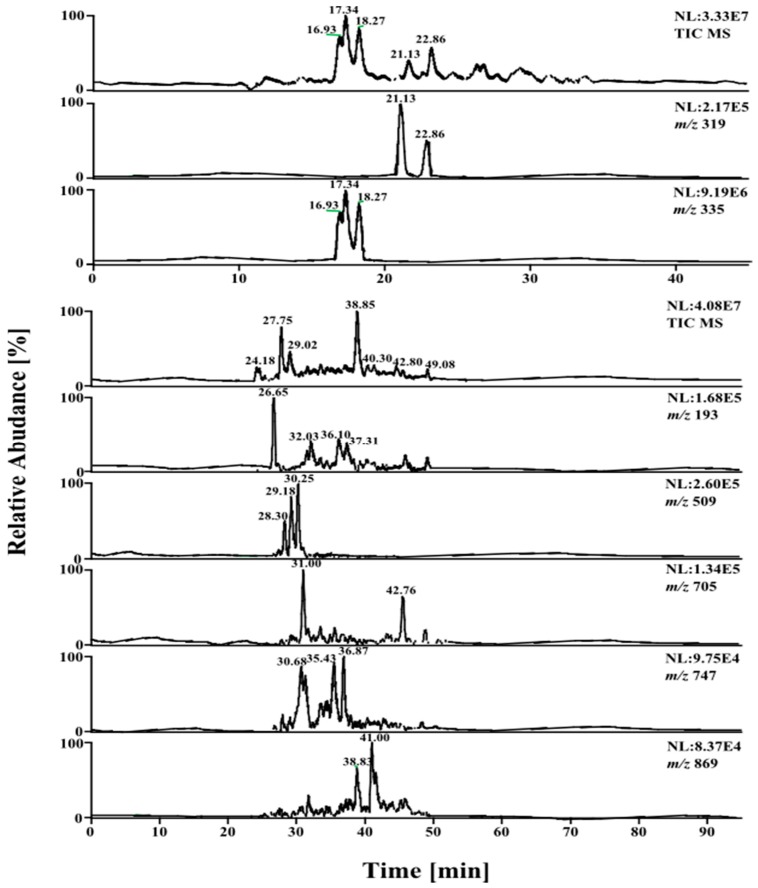
LC-ESI-MS-TIC and reconstructed ion chromatograms (RICs) (negative ion mode, base peak in the range 150–2000 *m*/*z*) of the phenolic compounds in male flowers of date palm.

MS experiments on LC-MS system coupled with an electrospray ion source and an ion trap mass spectrometer were performed in order to investigate the presence of different isobaric compounds and then to carry out a qualitative analysis of phenolic constituents occurring in 40% and 60% MeOH fractions from the extract of date palm male flowers. A full scan mass spectral data were obtained and reconstructed ion chromatograms (RICs) were extracted for each of the expected *m*/*z* values based on the molecular weights of the possible constituents (Figure 1).

The ESI-MS base peak chromatogram from the male flowers shows a relatively complex mixture containing peaks of phenolic acids, flavonoids, flavones and proanthocyanidins (monomers, dimers, trimmers and tetramers). Forty-eight phenolic compounds and five other organic acids were identified using fragmentation patterns observed in tandem mass spectra (Table 1). The isomers of caffeoylshikimic and coumaroylshikimic acid have been hypothesized using MS/MS spectra. Nineteen conjugated hydroxycinnamic acids with shikimic and quininc acids, six flavan-3-ol derivatives, two anthocyandin, and 21 flavonol and flavone derivatives were tentatively identified regarding to their fragmentation.

### 2.1. LC-ESI-MS and DFT Analysis of Hydroxycinnamic Acid Derivatives

**Figure 2:**
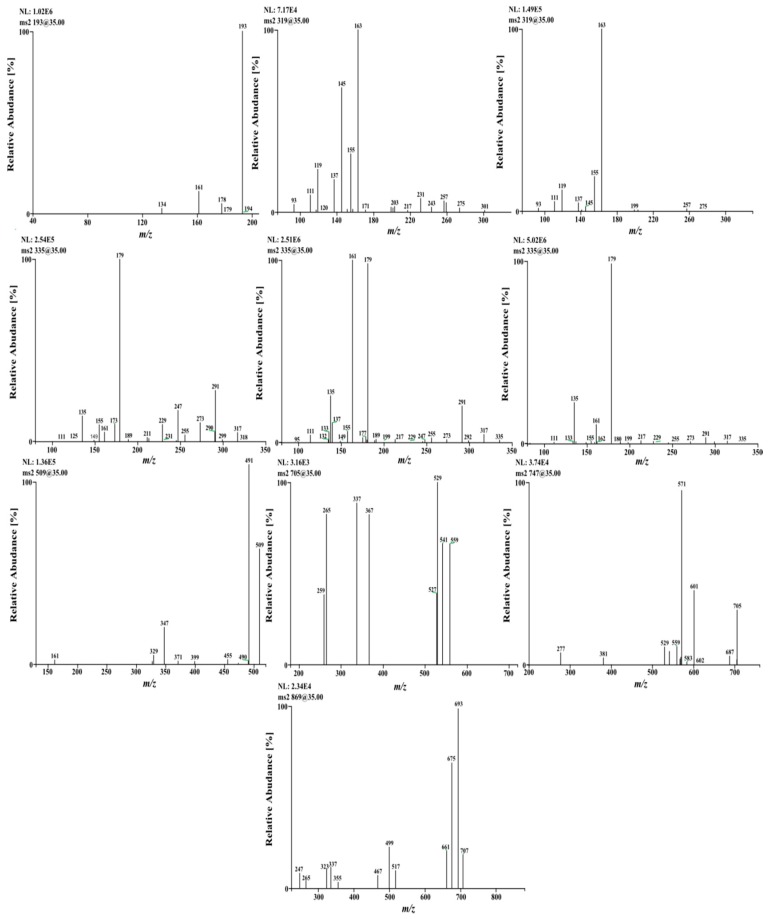
Product ion spectra of date palm male flowers components ([M − H]^−^): MS^2^ of regioisomers at *m*/*z* 319, dimers at *m*/*z* 335, at *m*/*z* 509, at *m*/*z* 705, at *m*/*z* 747, and 869.

**Scheme 1:**
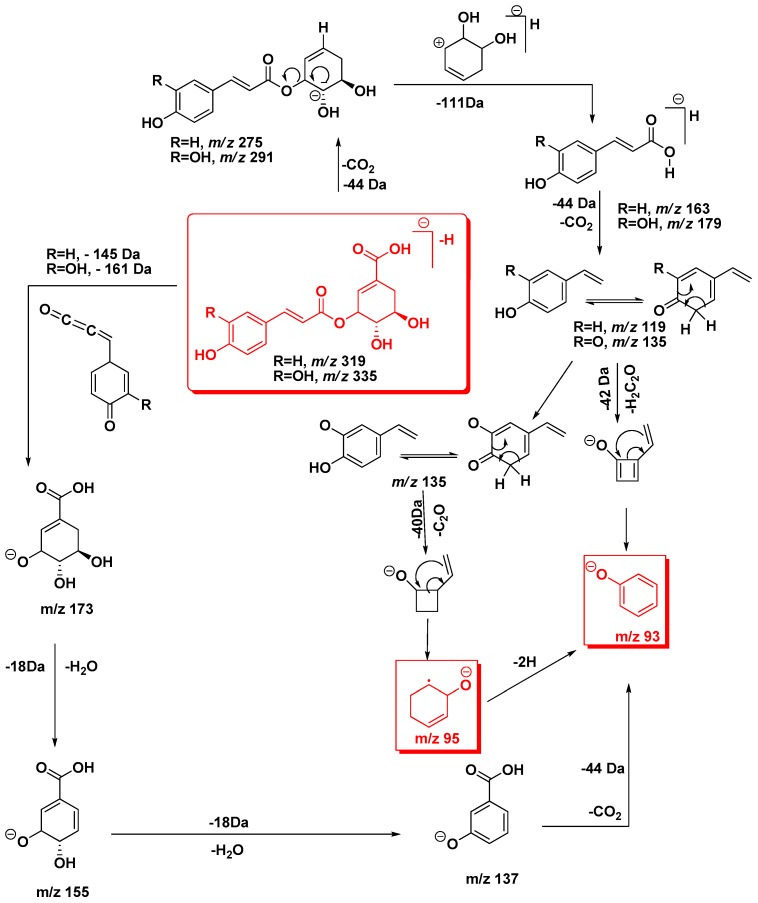

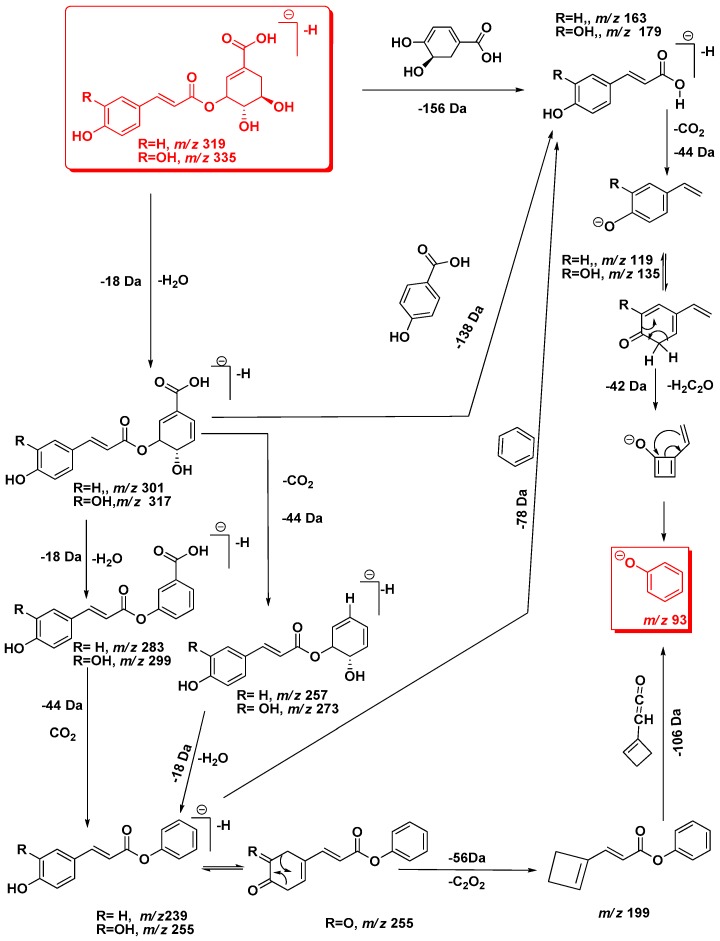
Proposed fragmentation pattern of *p*-coumaroylshikimic acid (R=H, *m*/*z* 319) and caffeoylshikimic acid (R=OH, *m*/*z* 335) isomers.

**Figure 3:**
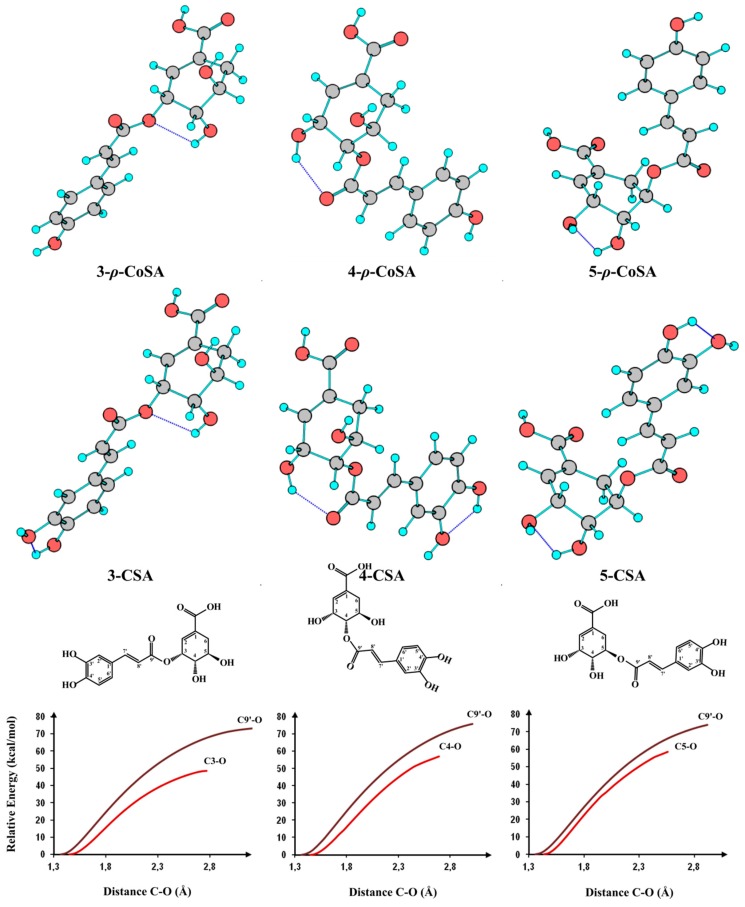
Optimized geometries of caffeoylshikimic acid isomers (CSA) and *p*-coumaroylshikimic acid isomers (*p*-CoSA) calculated at the B3LYP/6-31 + G(d,p) level, DFT/B3LYP relative energy along the scan of C–O distance.

Hypotheses of caffeoylshikimic acids (3-, 4- and 5-CSA) and *p*-coumaroylshikimic acids (3-, 4- and 5-*p*-CoSA) isomer’s were illustrated (Figure 2, Scheme 1). Moreover, DFT study was used to confirm their structures (Table 2, Figure 3). The product ion spectrum of the deprotonated compound **1** at *m*/*z* 341 [M − H]^−^ at retention time (Rt) 1.12 min shows the base peak at *m*/*z* 179 due to cleavage of hexoside moiety (162 Da) as well as ions at *m*/*z* 161 [M − H-hexose-H_2_O]^−^ , *m*/*z* 153 [M − H-hexose-HC≡CH-H_2_O]^−^, *m*/*z* 143 [M − H-hexose-2 × H_2_O]^−^. Based on the above arguments compound **1** was tentatively assigned as caffeic acid-*O*-β-hexoside [17].

Three large peaks for compounds **2**, **3** and **4** (Rt 16.93, 17.34 and 18.27 min, respectively) were predominated in the phenolic profile and exhibited a quasi-molecular ions at *m*/*z* 335 [M − H]^−^, which could correspond to caffeoylshikimic acid regioisomers (Figure 3). Caffeic acid can be esterified at positions 3, 4 or 5 on shikimic acid (dehydrated quinic acid) to give three positional isomers. The three precursor ions did not show any reproducible differences in their MS/MS spectra and contained the same main ions at *m*/*z* 179, 161 and 135, characteristic of caffeoyl moiety. On the other hand, the previous studies differentiated between them depending on the intensity of the base peak for each isomer [9].

Generally, after collision-induced dissociation, phenolic acids produce fragmentation patterns characterized by the loss of a CO_2_ (44 Da) from the carboxylic acid group. Due to this neutral loss, each tentative isomer of caffeoylshikimic acid produced the same [M − H-CO_2_]^−^ ion at *m*/*z* 291. Loss of cyclohexa-4-ene-1,2-diol of 111 Da by β-elimination produced the base peak at *m*/*z* 179 [M − H-44-C_9_H_7_O_4_]^−^ (Figure 2, Scheme 1). Subsequent cleavage of 44 Da from the ion at *m*/*z* 179 gave the ion at *m*/*z* 135, followed by the loss of 42 Da to produce the oxybenzen ion at *m*/*z* 93 [phenoxide]¯ from 3-caffeoyl and 5-caffeoylshikimic acid but for 4-caffeoylshikimic acid losing 40 Da due to cleavage of 95 Da [cyclohex-2-enolate]¯. Phenoxide molecule is more stable than cyclohex-2-enolate molecule and hence it was absent in both 3-CSA and 5-CSA. In addition, the product ion at *m*/*z* 179 produced through several cleavage patterns such as the loss of 156 Da ([M − H-156]^−^, [M − H-H_2_O-138]^−^) and 158 Da ([M − H-2 × H_2_O-CO_2_-78]^−^).

A DFT/B3LYP/6-31 + G(d,p) optimized scan for the C9’–O and C3–O bond length was performed to estimate the value of cleavage energy. The optimized values of C9′–O and C3–O bond length of 3-caffeoylshikimic acid were 1.37 and 1.45 Å, respectively (Figure 3). This difference was due to conjugation of C9′–O bond in the acid group (mesomeric effect). The cleavage of C9’-O bond required more energy than the cleavage of C3–O, C4–O and C5–O bonds. In case of 3-CSA, the calculated energy to cleave C9′–O bond was approximately 74 kcal·mol^−1^ (3.21 eV), whereas the energies required to cleavage C3–O, C4–O and C5–O bonds were 48, 58 and 56 kcal·mol^−1^ (2.08, 2.52 and 2.43 eV), respectively. The breakdown curve for the three regioisomeric caffeoylshikimate showed that the cleavage energy was C3–O < C5–O < C4–O [19]. Thus, cleavages of C3–O, C4–O and C5–O bonds were easier than the cleavage of C9′–O bond. For this reason, all isomers gave the ion peaks at *m*/*z* 179 and 161 but their intensities were different. The three isomers do not show any reproducible differences in their MS/MS spectra. The MS/MS spectra gave a clear knowledge to hypotheses fragmentation pattern of these isomers (Scheme 1). Based on the above arguments isomers were tentatively assigned as 3-, 4- and 5-caffeoylshikimic acids (3-CSA, 4-CSA and 5-CSA). 

**Figure 4:**
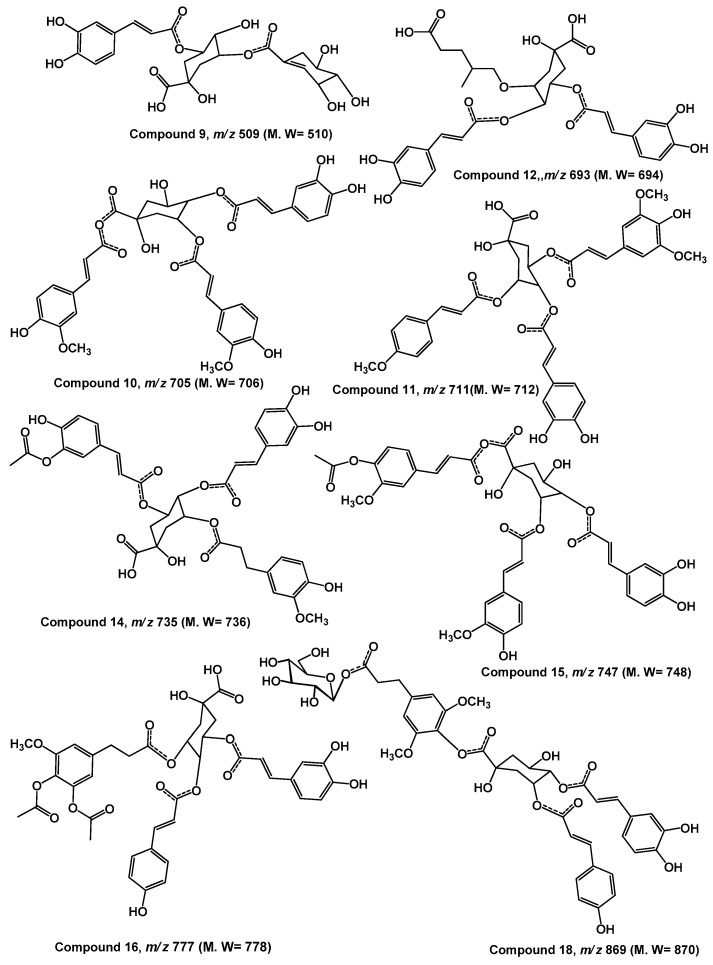
Proposed structures of the some phenolic compounds in *Phoenix dactylifera* male flowers.

Similarly, quasi-molecular ion *m*/*z* 319 forming compounds **5** and **6** at Rt 21.13 and 22.92 min, respectively, were tentatively corresponding to *p*-coumaroylshikimic acid (Figure 2, Table 1). These peaks have the same fragmentation spectra, established by the presence of ions at *m*/*z* 163, 145 and at *m*/*z* 119, which are characteristic for *p*-coumaroyl moiety. On the other hand, the base peak for each isomer was similar at *m*/*z* 163 but the intensity of the peak at *m*/*z* 145 showing a difference in each (Figure 2 and Figure 4 and Scheme 1). Based on the arguments presented above, the two isomers were tentatively assigned as 3 and 5-*p*-coumaroylshikimic acids (3-*p*-CoSA and 5-*p*-CoSA). Structures of all these isomers were optimized by means of the DFT/B3LYP method and their local minima on the singlet PES were studied. Relative energies as well as corrections of zero point (ZPE), thermal enthalpy and free energy were gathered in Table 2 and optimized geometries are given in Figure 3. The difference in energy between the regioisomers of caffeoylshikimic acids (CSA) was 0.3 kcal·mol^−1^. The isomers 3-CSA and 5-CSA were more stable than isomer 4-CSA by 0.3 kcal·mol^−1^. Moreover, there were similarity differences in energy between the isomers of *p*-coumaroylshikimic acids (*p*-CoSA) (Table 2). The relative free energy for each series of regioisomers has been calculated. The population of each isomer was carried out in the gas state at 298 K by using the Boltzmann population. The 3-CSA and 3-*p*-CoSA isomers were the most prevalent (67% and 76% respectively). The 5-CSA and 5-*p*-CoSA isomers population are 28% and 15%, respectively. We concluded that all regioisomers were existed, but the more prevalent regioisomers were (3-CSA and 3-*p*-CoSA). 

The product ion spectrum of the deprotonated compound **7** at *m*/*z* 339 (Rt 25.50 min) shows a base peak [M − H-44]^−^ at *m*/*z* 295 due to cleavage of neutral CO_2_ and at *m*/*z* 251 [M − H-2 × 44]^−^, due to cleavage of second neutral CO_2_. Another peak at *m*/*z* 179 [M − H-160]^−^, due to cleavage of caffeic acid moiety. Similar compound has been recorded in wine but the fragmentation pattern was different (at *m*/*z* 293 and 177), named as ethylcaffeoyl [20]. On the basis of the above arguments compound **7** was tentatively assigned as caffeoyl-2-hydroxyethane-1,1,2-tricarboxylic acid.

The quasi-molecular ion of the deprotonated compound **8** at *m*/*z* 193 [M − H]^−^ (Rt 26.71 min) showed the diagnostic product ion at *m*/*z* 178 [M − H-15]^−^ was due to cleavage of methyl group. Moreover, the product ion at *m*/*z* 134 [M − H-15-44]^−^ attributable to the sequential loss of CO_2_ and CH3. Based on the above arguments compound **8** was tentatively assigned as ferulic acid.

The quasi-molecular ion of the deprotonated compound **9** at *m*/*z* 509 [M − H]^−^ (Rt 30.12 min) showed the diagnostic product ion at *m*/*z* 353 [M − H-156]^−^ characterized for chlorogenic acid moiety and this was confirmed Losing of 172 indicated the presence of shikimate moiety [9]. In addition, it showed the presence of ion peaks at *m*/*z* 491 [M − H-H_2_O]^−^ due to loss 18 Da and at *m*/*z* 347 [M − H-162]^−^ due to cleavage of caffeoyl moiety as well as ion at *m*/*z* 329 [M − H-caffeoyl-H_2_O]^−^ which confirmed losing of caffeoyl moiety. The mass of this compound was tentatively assigned as caffeoylquinate shikmate derivative.

Table 1 and Figure 4 showed deprotonated peaks for shikimic and quininc acid derivatives conjugated with different cinnamate derivatives at *m*/*z* 705 (**10**), 675 (**11**), 693 (**12**), 559 (**13**), 735 (**14**), 747 (**15**), 777 (**16**), 711 (**17**) and 869 (**18**). The deprotonated ion peak at *m*/*z* 693 [M − H]^−^ for compound **12** (Rt 32.64) showed a product ion at *m*/*z* 353 [M − H]^−^ was characteristic for caffeoylquinic acid. The diagnostic product ions at *m*/*z* 499 and 337 were characterized to *p*-coumarylquinic acid moiety [9,10,19,21]. Moreover, the product ion at *m*/*z* 499 [M − H-114]^−^ was probably due to loss of 5-hydroxy-4-methylpentanoic acid. The mass of this compound was tentatively assigned as 5-*O*-*p*-coumaroyl-4-*O*-caffeoyl-4-methylpentanoic acid-5-hydroxy-3-quinate. The deprotonated ion peak at *m*/*z* 747 [M − H]—for compound **15** (Rt 35.46) showed a product ion at *m*/*z* 705 [M − H-42]^−^ was due to loss of acyl moiety, product ion at *m*/*z* 571 [M − H-176]^−^ due to loss of ferulic moiety which probably linked at C7 of quinic acid. The presence of the product ion at *m*/*z* 529 indicated the presence of 3-feruloyl-4-caffeoylquinic acid moiety. Comparison the fragmentation patterns of compounds **10** and **15** indicated that they are similar except compound **15** has more acyl group. The proposed structure for compound **10** was 3,7-*O*-diferuloyl-4-*O*-caffeoyl quinic acid and for compound **15** was 3-*O*-feruloyl-7-*O*-acyl-feruloyl-4-*O*-caffeoyl-quinic acid [21].

The precursor ion of an unique compound **19** at *m*/*z* 418 [M − H]^−^ eluting at Rt 67.66 min. The product ion at *m*/*z* 163 [M − H-18]^−^, due to loss of 256 Da which may be due to the cleavage of 16-hydroxypalmatic acid moiety. The presence of product ions at *m*/*z* 163, 145, 119 and 93 are characteristic for *p*-coumaroyl moiety. 16-hydroxy palmitic acid is a hydroxyl fatty acid derivative and it was conjugated with *p*-coumaric acid. Based on the above arguments compound **19** was tentatively assigned as *p*-coumaroyl-*O*-16-hydroxypalmitic acid isomer [19].

### 2.2. LC-ESI-MS of Fatty Acids

MS showed several saturated polyhydroxy fatty acid isomers (Table 1). The product ions spectrum of the deprotonated compounds **21**, **22**, **23** and **24** (at *m*/*z* 329, 327, 347 and 333, respectively) were tentatively assigned as hydroxy-octadecadienoic acid derivatives [22,23,24,25].

### 2.3. LC-ESI-MS Analysis of Proanthocyanidins Derivatives

The obtained *m*/*z* values evidenced the presence of six flavan-3-ol derivatives belonging to catechin and gallocatechin series. Proanthocyanidins are formed from the condensation of monomeric units and may differ in the position and configuration of their monomeric linkage. ESI-MS fingerprint obtained from *P. dactylifera* extract exhibited a complex mixture containing molecular ions for proanthocyanidin monomers, dimers, trimmers and tetramers.

The two deprotonated ions at *m*/*z* 289 with different retention times resulted from the diastereoisomers (catechin and epicatechin). MS/MS spectrum pattern of the ion peaks at *m*/*z* 289 exhibited the major product ions at *m*/*z* 109, 125, 137, 151, 165, 179, 205, 245, 247 and 271. The product ion at *m*/*z* 271 ion was derived from the loss of a water equivalent (18 Da) and the product ions at *m*/*z* 245 and 247 were due to the loss of 42 and 44 Da (HC≡C–OH, CH_2_=CH–OH). The product ion at *m*/*z* 165 [M − H-125]^−^ may be resulted after the elimination of the ring A from it by the HRF fission [11]. The ion with *m*/*z* 125 [M − H-165]^−^ confirmed the HRF fission. The ion product at *m*/*z* 137 was resulted from an RDA of ring C that was confirmed by the presence of product ion at *m*/*z* 151 [11]. The diagnostic product ion at *m*/*z* 179 [M − H-110]^−^ was due to the loss of dihydroxybenzene moiety which was detected by the presence of product ion at *m*/*z* 109 [M − H-179]^−^. The product ion at *m*/*z* 167 [M − H-122]^−^ probably formed by BFF fission [11]. The ESI-MS/MS spectral data of afzelechin (at *m*/*z* 273) and gallocatechin (at *m*/*z* 305) derivatives were similar of catechin and epicatechin [24]. 

In Table 1, the spectra of date palm male flowers PAC dimers are represented by the two B-type compounds with quasi-molecular ions at *m*/*z* 577 and 593 [23,24]. Their MS/MS spectra elucidated that the relative abundance of major product ions (e.g., 287, 289, 305, 407, 409, 417, 421, 425, 433, 439, 441, 449) varied, indicating that they may represent different isomeric forms, arising from the different linkage of monomeric flavan-3-ol units [11].

**Figure 5:**
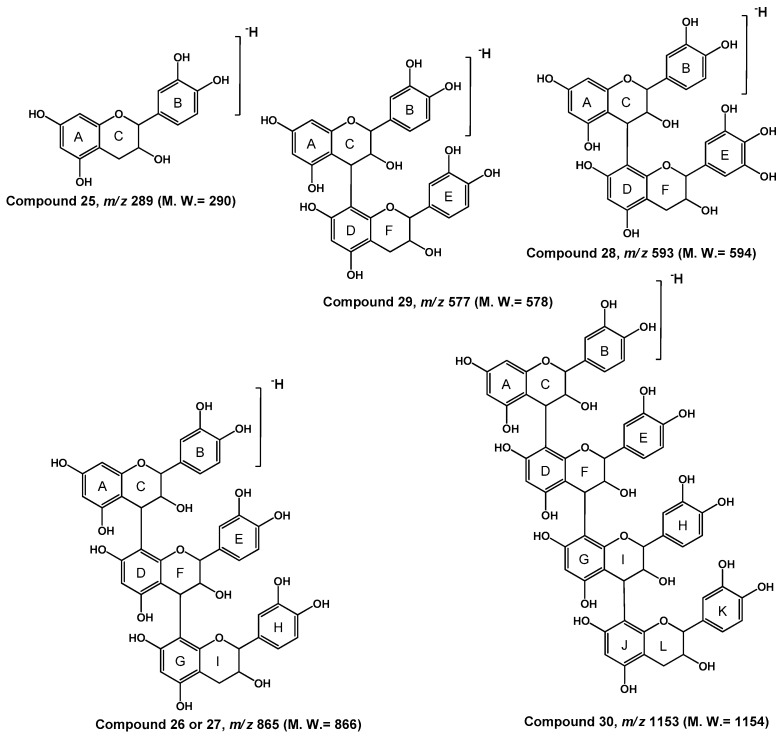
Structure of some phenolic compounds in male flowers of *Phoenix dactylifera* male flowers.

The quasi-molecular ion of compounds **26** and **27** at *m*/*z* 865 (Rt 12.20 and 13.64 min.) exhibited the diagnostic product ion at *m*/*z* 713 [M − H-152]^−^ due to the retro-Diels-Alder (RDA) reaction and loss of the 1, 3 B group (Figure 5). The RDA rearrangement was found to dominate, particularly on the C-ring of catechin and epicatechin derivatives, in which two possible product ions can be created by a 152 Da loss of a B-ring or a 136 Da removal of an A-ring [11]. The RDA reaction could occurred on the top unit or on the base unit of the trimer. Moreover, RDA yielded the diagnostic product ion at *m*/*z* 577 [M − H-152-136]^−^ due to the loss of C_7_H_4_O_3_, probably by the cleavage of 3, 4 double bond and A-ring. The diagnostic product ion of the deprotonated ion at *m*/*z* 577 gave the base peak at *m*/*z* 407 [M − H-152-136-170]^−^ due to the RDA fragmentation and loss of the 1, 3 E group and loss of water from I-ring. The loss of 126 Da indicated that the A ring of top unit had a 1,3,5-trihydroxybenzene structure. HRF yielded the product ion at *m*/*z* 577 [M − H-126-162]^−^ which was due to the loss of C_7_H_4_O_3_, by cleavage of 3, 4 double bond and A-ring [12]. Moreover, formation of the diagnostic product ion at *m*/*z* 577 indicated that the presence of two hydroxyl groups located at C-3′ and C-4′ of the B ring. Hence, the top, middle and base units of this trimer were identified as catechin. Other minor product ions were observed at *m*/*z* 847 [M − H-18]^−^ due to the loss of water from the trimer, at *m*/*z* 577 [M − H-288]^−^ and at *m*/*z* 271 [M − H-290]^−^ resulting from the cleavage of the ion at *m*/*z* 407 through the QM mechanism. The proanthocyanidins that consist exclusively of [epi]catechin are called procyanidins and the ion at *m*/*z* 865 and *m*/*z* 577 are indicative of the B-type procyanidintrimer, i.e., procyanidin containing singly linked units. However, as with other MS techniques, no differentiation between stereoisomers is possible and no acquaintance about the position and stereochemistry of the inter-flavanoid linkage (4→6 or 4→8) is available. It has been revealed that procyanidins with 4→8 linkages are stereochemically favored, this trimer was tentatively established as (E)catechin–(E)catechin–(E)catechin [24,25,26].

Compound **28** with the quasi-molecular ion at *m*/*z* 593 shows the diagnostic product ion at *m*/*z* 441, which was originated after neutral loss of 152 Da (RDA) due to cleavage of ring B from the flavan-3-ol through RDA reaction of ring C. This loss indicates that ring B of the top unit had two hydroxyl groups. RDA reaction was confirmed by the presence of the diagnostic product ion at *m*/*z* 305 [M − H-441-36]^−^, due to loss of the A ring. The presence of two ions: *m*/*z* 467 [M − H-126]^−^, due to HRF fission on the top unit of the dimer, and *m*/*z* 449 [M − H-126-18]^−^, attributable to the sequential loss of water from the ring F, indicated that the A ring of the top unit had a 1,3,5-trihydroxybenzene moiety. Formation of the ion at *m*/*z* 449 indicated that the two hydroxyl groups were located at 3′ and 4′ positions. Hence, the top unit of this dimer was identified as catechin derivative. Another minor ion at *m*/*z* 407 [M − H-168-18]^−^ was due to RDA reaction of ring F, indicated that ring E of the base unit had three hydroxyl groups located at 3′, 4′ and 5′. Hence, the base unit of this dimer was tentatively established as gallocatechin derivative. This sequence was confirmed through QM cleavage that produced two ions at *m*/*z* 305 and 289. The connection sequence of this dimer is proposed to be (E)catechin–(E)gallocatechin. 

After neutral losing of a 152 Da, the deprotonate peak ion at *m*/*z* 577 give the product ion at *m*/*z* 425 due to the cleavage of ring B from the flavan-3-ol through RDA reaction of ring C. Loss of 152 Da indicates that ring B of the top unit has two hydroxyl groups. The product ion at *m*/*z* 425 was due to loss of 18 Da (H_2_O), most likely from the free 3-OH, to give a stable product ion at *m*/*z* 407 (RDA). The *m*/*z* 451 ion was formed after HRF on the top unit of the dimer. HRF on the base unit of the dimer was not suitable for the same reason as RDA. Loss of 126 Da indicates that the A ring of the top unit has a 1,3,5-trihydroxybenzene structure. Moreover, formation of the diagnostic product ion at *m*/*z* 451 was due to the cleavage of two hydroxyl moieties at the position 3′ and 4′ of the B ring. The top unit of this dimer was tentatively identified as catechin derivative. Because the chirality of C-3 on the flavan-3-ols cannot be differentiated by MS, catechin elucidates either catechin or epicatechin. The connection sequence of this dimer has been derived to be catechin-catechin [24,25,26]. Identification of this sequence was confirmed by the diagnostic product ions at *m*/*z* 289 [M − H-288]^−^ and *m*/*z* 287 [M − H-290]^−^. These two product ions were formed from the top and the base unit after QM cleavage of the interflavan bond. Therefore, compound **29** was identified to be a singly linked proanthocyanidin dimer. The connection sequence of it has been established to be (E)catechin–(E)catechin.

The quasi-molecular ion [M − H]^−^ at *m*/*z* 1153 [3 × 288 + 289-H]− was similar with those of (E)catechin polymers. As shown in Table 1, the fragmentation pattern of compound **30** at *m*/*z* 1153 gave product ions at *m*/*z* 865 [M − H-288]^−^ due to the quinine methide reaction (QM1) of the top unit. Moreover, the diagnostic product ion at *m*/*z* 701 [M − H-288-164]^−^ was due to HRF reaction of the inter-flavone unite ([M − H-288-C_9_H_8_O_3_]^−^), *m*/*z* 575 [M − H-288-C_9_H_8_O_3_-126]^−^ was due to the loss of a tri-hydroxybenzene, probably by the cleavage of D-ring from the middle unit. In addition, the diagnostic product ion at *m*/*z* 575 [M − H-2 × 289]^−^, was due to the quinine methide reactions (QM1, QM4) of the top and lower units. The product ion at *m*/*z* 449 [M − H-575-126]^−^, was due to loss of another trihydroxybenzene, probably by elimination of D-ring or G-ring from one of the two middle units. The presence of the diagnostic product ion at *m*/*z* 425 [M − H-575-C_6_H_6_O_3_-H_2_O]^−^, due to the BFF mechanism from the lower unit of the two intermediate 3-flavonol units (Figure 5). Its structure was tentatively established as (E)catechin–(E)catechin–(E)catechin–(E)catechin. 

### 2.4. LC-ESI-MS Analysis of Flavonol and Flavone Derivatives

Flavonoid glycosides were previously reported in date palm male flowers [8]. 21 flavonol and flavone derivatives were tentatively identified regarding to their fragmentation patterns (Table 1). Fragmentation pattern of compounds **34**, **35**, **46** and **48** were contained diagnostic ions at *m*/*z* 301 suggesting quercetin and quercetin derivatives [27]. Similarly, fragmentation spectra of compounds **38**, **39**, **43**, **44**, **45** and **50**, also show diagnostic product ions for orientin or isoorientin derivatives at *m*/*z* 299. Deprotonated ions of compounds **37** had the same fragment ions *m*/*z* 285 corresponding to kaempferol in their MS/MS spectra. The ion peak for compound **53** at Rt 35.84 min gave [M − H]^−^ ion at *m*/*z* 301 [M − H]^−^, corresponding to quercetin moiety. This was confirmed by the presence of peaks at *m*/*z* 257 [M − H-44]^−^, due to loss of CO_2_ molecule and at *m*/*z* 229 [M − H-18-44]^−^, due to loss of CO_2_ molecule.

## 3. Material and Methods

### 3.1. Plant Materials

Fresh male flowers of *Phoenix dactylifera* were collected from Al-Rass governor (Qassim, Saudi Arabia) in March 2013. The plant material was identified by Prof A. I. Hamed according to Täckholm (1974) and the voucher specimens No. 21 were deposited in the Natural Products Laboratory, Chemistry Department, College of Science and Arts (Al-Rass, Saudi Arabia).

### 3.2. Extraction and Fractionation

The fresh male flowers (100 g) were exhaustively extracted with 80% MeOH (1.5 L) for 6 h. in a Soxhlet apparatus at 50 °C. The crude extract was concentrated under reduced pressure to a syrupy consistency (20 g). 15 g of the crude extract was dissolved in a small quantity of H_2_O and was loaded on a water preconditioned short C18 column (6 cm × 10 cm, LiChroprep_RP-18, 40–60 µm, Merck, Germany). Six fractions (1000 mL each) were collected: 100% H_2_O, 40% MeOH (phenolics fraction), 60% MeOH (phenolics fraction), 80% MeOH (saponins fraction) and 100% MeOH. All fractions were separately concentrated under reduced pressure to obtain crude fractions. The phenolic fractions (0.1 mg) was separately dissolved in 1 mL of methanol, and diluted 1:10 with same solvent before analysis of 20 μL in the chromatographic systems.

### 3.3. High-Performance Liquid Chromatography and ESI-Mass Spectrometry of Phenolic Fraction

The 40% and 60% crude methanolic fractions were separately analyzed by an HPLC-ESI-ITMS system using a Thermo LCQ Advantage Max ion trap mass spectrometer (Thermo Fisher Scientific, 168 Third Avenue, Waltham, MA, USA) equipped with Xcalibur software. LC separation was carried out on a Thermo Surveyor HPLC system and a Waters Symmetry C_18_ column (5 µm, 2.1 mm × 150 mm; Waters, MA, USA). Analytes were separated using a 45 min long linear gradient from 5% to 30% of mobile phase B (acetonitrile containing 0.03% (*v*/*v*) formic acid) in mobile phase A (HPLC-grade water containing 0.03% formic acid). The flow rate was 0.4 mL/min, and column temperature was 50 °C.

The flow from the LC system was inserted into the ESI ion source (negative ion mode). The ion source was operated with a capillary temperature 230 °C, capillary voltage −47 V, spray voltage 3.9 kV and tube lens offset −50 V. The flow rate of sheath gas (N_2_) was 70 (arbitrary units) and the flow rate of auxiliary gas (N_2_) was 10 (arbitrary units). The scan range was *m*/*z* 150–2000, the duration for each injection was 150 ms and the number of micro-scan was two. The first scan step was a full-scan mass spectrum to obtain data on anions in the specified scan range. The collision energy of the second scan step (MS/MS) was 35%. The resulting product ions [M − H]^−^ are examined in a second mass measurement step (MS^2^) [13,28]. 

### 3.4. DFT Study

Relative stabilities of these isomers were optimized using the DFT approach implemented in the GAUSSIAN09 series of programs [29]. A 6–31 + G(d,p) basis set was employed for each atom. The potential energy surfaces (PES) was described by using the B3LYP hybrid functional [30]. The analytic gradients were employed in order to optimize the geometries of the isomers. In order to identify the local minima and to estimate the corresponding zero-point vibrational energy (ZPE) [31,32], the frequencies of the isomers were calculated. The thermodynamic quantities (relative energies, corrections of zero point energy, thermal enthalpy, and free energy differences) at 298 K in the gas phase of caffeoylshikimic acids (3-, 4- and 5-CSA) and *p*-coumaroylshikimic acids (3-, 4- and 5-*p*-CoSA) were done. The relative populations of the different isomers were calculated from Boltzmann’s distribution using the calculated values of Gibbs free energy: $\frac{P_{i}}{P_{j}}=\;e^{\frac{G_{j}^{{}^{\circ}}-G_{j}^{{}^{\circ}}}{k_{B}T}}$, where $P_{n}$ is the probability of state n, $G_{n}^{{}^{\circ}}$ is the free energy of state n with n=(i,j), $k_{B}$ is the Boltzmann constant, T is the temperature of the system.

## 4. Conclusions

The analytical procedure elucidated in this work proved a rapid, simple method to simultaneously extract phenolic acids, proanthocyanidins and flavonoids. Oligomers and polymers detection is important for plant scientists; however, the constituent units and the connection sequence of low oligomers (DP < 5) identification are crucial for the nutritionists who are interested to study their possible human health and bioavailability effects. This qualitative analysis provided a valuable fingerprint of the main metabolites occurring in *P. dactylifera* male flowers. This is the first phytochemical analysis has been done from male flowers. The varieties of the structures of B-type proanthocyanidin, monomers, dimers, trimmers and tetramers were revealed by HPLC-ESI-MS/MS studies. The identification of B-type linkages using LC-MS/MS alone eliminates a number of tedious separation steps. The data obtained in our research shows that *P. dactylifera* male flowers could be a good source of hydrolysable proanthocyanidins, flavonoids glycosides and phenolic cinnamate derivatives, therefore it can be considered as a rich source of catechin and gallocatechin derivatives. Qualitative analysis results confirmed that male flowers of *P. dactylifera* obtained during flower production could represent an interesting source of phenolic compounds, with respect to the high variety of compounds showed by the metabolic profiling analysis presented in this experimental work.

## Figures and Tables

**Table 1:** Characterization of polyphenolic compounds in male flowers of date palm using LCESI-MS/MS in negative ion mode.

Comp.	Rt	Tentative Identification	MW	[M − H]^−^	Major Fragments (*m*/*z*)
**Hydroxycinnamates**					
1	1.12	Caffeoyl-*O*-hexoside	342	341	179 (**100**), 161, 153, 143
2	16.93	5-*O*-caffeoylshikimic acid (5-CSA)	336	335	317, 299, 291, 219, 273, 255, 179 (**100**), 161, 155, 137, 135, 93
3	17.34	4-*O*-caffeoylshikimic acid (4-CSA)	336	335	317, 291, 219, 273, 255, 179 (**100**), 161 (**100**), 155, 137, 135, 95
4	18.27	3-*O*-caffeoylshikimic acid (3-CSA)	336	335	317, 299, 291, 219, 273, 255, 179 (**100**), 161, 155, 137, 135, 93
5	21.13	5-*O*-*p*-coumaroylshikimic acid (5-*p*-CoSA)	320	319	301, 275, 257, 243, 231, 217, 203, 171, 163 (**100**), 155, 145, 137, 119, 93
6	22.92	4-*O*-*p*-coumaroylshikimic acid (3-*p*-CoSA)	320	319	275, 257, 199, 163 (**100**), 155, 145, 137, 119, 93
7	25.50	caffeoyl-2-hydroxyethane-1,1,2-tricarboxylic acid	340	339	295, 251, 179 (**100**)
8	26.71	Ferulic acid	194	193	178, 161, 134
9	30.12	caffeoylquinate shikmate derivative	510	509	491 (**100**), 473, 455, 389, 371, 347, 329, 161
10	31.00	3,7-*O*-diferuloyl-4-*O*-caffeoyl quinic acid	706	705	687, 663, 645, 559, 541, 529 (**100**), 527, 511, 483, 405, 337, 265, 259
11	32.22	Feruloyl-*O*-*p*-coumaroyl-*O*-caffeoylshikimic acid	676	675	643, 627, 603, 588, 513 (**100**), 495, 493, 463, 431, 375, 339, 313, 295 (**100**)
12	32.64	5-*O*-*p*-coumaroyl-4-*O*-caffeoyl-4-methylpentanoic acid-5-hydroxy-3-quinate	694	693	663, 499, 353, 345, 337, 221
13	32.80	1-*O*-caffeoyl-3-*O*-sinapoylquinic acid	560	559	381
14	34.79	Feruloyl-*O*-sinapoyl-*O*-caffeoylquinic acid	736	735	693, 675, 559, 541, 527, 517, 513, 481, 479, 455, 323, 305
15	35.46	3-*O*-feruloyl-7-*O*-acyl-feruloyl-4-*O*-caffeoyl-quinic acid	748	747	729, 705, 687, 591, 583, 574, 571 (**100**), 573, 559, 547, 529, 409, 381, 357, 333, 277
16	37.50	(*E*)-3-(3,4-diacetoxy-5-methoxyphenyl)acroyl-4-*O*-*p*-coumaroyl-5-*O*-caffeoylquinic acid	778	777	759, 735, 717, 693, 675, 615, 601, 559, 541, 527, 517, 513, 499, 481, 479, 455, 439, 425, 337,323, 305, 279, 271, 247, 245, 217
17	37.61	Tri-caffeoylquinic acid	712	711	679, 667, 635, 535, 387, 355, 353
18	40.95	3-*O*-*p*-coumaroyl-4-*O*-hydrosinapoyl-*O*-hexoside-5-*O*-caffeoylquinic acid	870	869	707, 693 (**100**), 675, 661, 499, 413, 355, 337, 323, 265, 247
19	67.66	*p*-coumaroyl-*O*-16-hydroxypalmatic acid	419	418	163, 145, 119, 93
**Other organic acids**					
20	15.81	Ascorbic acid	176	175	157
21	25.51	Trihydroxy-octadecanoic acid isomer	330	329	314 (**100**), 135
22	29.05	Trihydroxy-octadecadienoic acid isomer	328	327	206
23	30.39	Pentahydroxy-octadecanoic acid isomer	348	347	329, 311, 304, 193, 161
24	32.42	Trihydroxyoctadecanoic acid sulphate	334	333	315 (**100**), 235, 151, 97
**Flavan-3-ols**					
25	11.63	(E)Catechin ^a^	290	289	271, 247, 245 (**100**), 231, 221, 205, 179, 167, 151, 139, 125, 109
26	12.20	(E)Catechin–(E)Catechin–(E)Catechin ^a^	866	865	847, 739, 713, 647, 627, 617, 577, 575 (**100**), 559, 515, 449, 477, 404, 327, 287
27	21.42	(E)Catechin–(E)Catechin–(E)Catechin ^a^	866	865	847, 739, 713, 647, 627, 617, 577, 575 (**100**), 559, 515, 449, 477, 404, 327, 287
28	27.56	(E)Catechin–(E)Gallocatechin ^a^	594	593	575, 557, 549, 513, 487, 473, 449, 441, 407, 343, 305 (**100**), 289, 187
**Flavan-3-ols**					
29	27.61	(E)Catechin–(E)Catechin ^a^	578	577	435, 425, 407, 289 (**100**), 287
30	30.54	(E)Catechin–(E)Catechin–(E)Catechin–(E)Catechin^a^	1154	1153	1065, 1043, 1027 (**100**), 983, 947, 865 (**100**), 711, 701, 693, 651, 605, 575, 549, 425, 407, 289
**Anthocyanidins**					
31	25.19	Cyanidin	287	286	271, 225, 151 (**100**), 135, 125, 107
32	30.27	Pelargonidin dimethyl derivative	301	300	271 (**100**), 243, 163
**Flavonols and flavones**					
33	21.23	Dihexosylisorhamentin	640	639	611, 477, 315 (**100**), 300, 280, 270, 255, 242, 214, 205
34	21.53	Hexosylquercetin	464	463	343, 301(**100**), 217
35	21.83	Dihexosylquercetin	626	625	463, 314, 301 (**100**)
36	23.54	Gliricidin or 3-*O*-methylorobol	300	299	284 (**100**), 272, 271, 255, 254
37	24.21	Rhamnosylhexosyl luteolin	594	593	575, 447 (**100**), 429, 327, 285 (**100**), 257, 241, 215, 195, 177
38	24.47	Isorhamentin ^b^	315	314	299 (**100**), 286, 285, 271, 255, 243, 230
39	24.67	Rhamnosylhexosyl methyl quercetin	624	623	608, 591, 519, 477, 459, 339, 315 (**100**), 299
40	25.14	Dihydrokaempferol	288	287	272, 225, 160, 151, 139, 125, 107
41	25.88	Isorhamentin hexoside	478	477	409, 357, 329, 315 (**100**), 300, 271, 243, 173
42	26.65	Kaempferol	286	285	257, 241, 217,213, 197
43	26.76	Isorhamentin ^b^	315	314	299, 286 (**100**), 285, 271
44	26.85	Orientin or Isoorientin	448	447	299 (**100**), 284
45	28.22	(Orientin or Isoorientin)-sulphate	528	527	447, 299
46	28.90	Quercetin rutinoside	610	609	591, 465, 463, 373, 343, 301 (**100**), 287, 271, 255, 226, 179
47	29.73	Gliricidin or 3-*O*-methylorobol	300	299	284 (**100**), 272, 271, 255, 254, 202
48	29.99	quercetin acetyl-hexoside	506	505	463, 301 (**100**), 300
49	31.54	Hexosyl-acyl-isorhamnetin	520	519	459, 451, 357, 315 (**100**), 314, 299, 285, 243, 204, 187, 151
50	31.64	Chrysoeriol hexoside	462	461	446, 299
51	34.08	Rhamnosyl-hexosyl-acyl-quercetin	650	649	607, 589, 503, 485, 407, 341, 299 (**100**), 284
52	34.90	Rhamnosylhexosyl-methyl-quercetin	612	611	594, 521, 491, 449, 327, 286
53	35.84	Quercetin	302	301	273, 257, 229, 215 (**100**), 211, 193, 179, 169, 151, 111, 107

^a^: indicates there are two possibilities ‘‘catechin or epicatechin; gallocatechin or epigallocatechin”; ^b^: indicates there are two possibilities; Base peaks between parenthesis and bold.

**Table 2:** Relative energies (kcal·mol^−1^) and populations at 298 K in the gas phase of caffeoylshikimic acid (CSA) and *p*-coumaroylshikimic acid (3-*p*-CoSA)isomer’s calculated at the B3LYP/6-31 + G(d,p) level.

Compound	ΔE	ΔE + ZPE	ΔH	ΔG	Population (%)
3-CSA	0.0	0.0	0.0	0.0	67
4-CSA	0.3	0.4	0.2	1.5	5
5-CSA	0.1	0.0	0.1	0.5	28
3-*p*-CoSA	0.0	0.0	0.0	0.0	76
4-*p*-CoSA	0.3	0.5	0.3	1.5	9
5-*p*-CoSA	0.0	0.2	0.1	0.9	15
